# Efficacy and safety of camrelizumab for the treatment of cervical cancer: a systematic review and meta-analysis

**DOI:** 10.3389/fonc.2024.1526103

**Published:** 2024-12-24

**Authors:** Xiaodong Mi, Fei Tuo, Tong Lin

**Affiliations:** Department of Obstetrics and Gynecology, First Affiliated Hospital of Jishou University, Jishou, Hunan, China

**Keywords:** camrelizumab, cervical cancer, PD-1, ICIS, immunotherapy

## Abstract

**Background:**

Cervical cancer (CC) is a prevalent malignancy in women and ranks fourth in global cancer-related mortality. The prognosis for women with metastatic or recurring cervical cancer is unfavorable. Camrelizumab is a humanized high-affinity IgG4-kappa monoclonal antibody targeting programmed cell death 1 (PD-1), which has been progressively documented as a therapy for advanced cervical cancer with good result metrics. Nonetheless, a comprehensive investigation of Camrelizumab’s efficacy in treating cervical cancer has yet to be conducted.

**Methods:**

We conducted a search across PubMed, Ovid Medline, Embase, Web of Science, Cochrane Library, Scopus, ProQuest, CNKI, Wan Fang, VIP database, and CBMdisc, restricting the establishment date of the databases to October 2024. The ROBINS-I Scale was utilized to evaluate the methodological quality of the included studies. Furthermore, information about CR, PR, SD, PD, ORR, DCR, median OS, median PFS, adverse events (AEs), and other relevant data was obtained. A meta-analysis was performed utilizing a random-effects model and effect size for illness.

**Results:**

This meta-analysis included six trials, including 238 people with CC. The aggregated outcomes for patients were as follows: CR (0.097, 95% CI: 0.032-0.186), PR (0.465, 95% CI: 0.291-0.638), SD (0.264, 95% CI: 0.124-0.403), PD (0.174, 95% CI: 0.051-0.296), ORR (0.577, 95% CI: 0.354-0.799), DCR (0.784, 95% CI: 0.652-0.916), AEs (all grades: 0.836, 95% CI: 0.629-1.000, ≥grade III: 0.472, 95% CI: 0.111-0.834). The predominant treatment-related adverse events included anemia (≤grade II: 0.295, 95% CI: 0.187-0.402; ≥grade III: 0.124, 95% CI: 0.018-0.230), elevated aspartate aminotransferase (≤grade II: 0.196, 95% CI: 0.013-0.380; ≥grade III: 0.030, 95% CI: 0.007-0.053), neutropenia (≤grade II: 0.206, 95% CI: 0.150-0.261; ≥grade III: 0.114, 95% CI: 0.066-0.162), thrombocytopenia (≤grade II: 0.295, 95% CI: 0.187-0.402), and fatigue (≤grade II: 0.174, 95% CI: 0.046-0.303).

**Conclusions:**

This meta-analysis demonstrates that camrelizumab is efficacious and well-tolerated in patients with cervical cancer.

**Systematic review registration:**

https://www.crd.york.ac.uk/prospero/, identifier CRD42024527065.

## 1 Introduction

The World Health Organization (WHO) has estimated that in 2020 there were approximately 604,127 cases of cervical cancer and 341,831 deaths globally, with an incidence of 13.3 cases per 100,000 women-years and a mortality rate of 7.2 deaths per 100,000 women-years (1). CC represents the fourth most common cancer among women, with a significant prevalence among younger age groups. In regions with developing economies, mortality rates linked to delayed diagnosis have significantly risen due to inadequacies in the organization of screening programs (2). Approximately 83% of new CC diagnoses and 88% of related deaths occur in low- and middle-income countries. In a total of 36 countries, including those in sub-Saharan Africa, Latin America, and India, CC is the leading cause of death through cancer-related causes (3). Persistent infection with high-risk human papillomavirus (HR-HPV) is strongly linked to cervical cancer. HR-HPV is crucial in advancing early-stage cervical precancerous lesions and accelerating the evolution of CC (4). The World Health Organization (WHO) has established a global plan to accelerate the elimination of CC. This comprehensive plan includes three primary interventions: immunization, early screening, and timely treatment. Although the incidence of cervical cancer has decreased in developed nations recently, there is a noticeable trend toward an earlier age of onset (5). Early-stage cervical cancer may be challenging to identify during standard gynecological assessments owing to modest or ambiguous clinical presentations. As the condition advances, patients may encounter contact vaginal bleeding, atypical bloody leucorrhea, heightened leucorrhea, irregular vaginal bleeding, or postmenopausal vaginal hemorrhage. Advanced cervical cancer may result in excessive vaginal bleeding along with a watery or rice soup-like discharge. Moreover, tumors encroaching onto adjacent organs may result in specific symptoms, such as hematuria when the bladder is involved and hematochezia when the rectum is affected. There is considerable inconsistency between regional guidelines with regard to the recommended treatment options for patients at different stages of the disease. The majority of recommended treatment options for patients with early-stage cervical cancer are radical hysterectomy and systematic lymph node dissection (2). For patients diagnosed with an advanced stage of cervical cancer at their initial visit, or for patients who have experienced recurrence following surgical treatment for early-stage cervical cancer, the recommended treatment options are palliative radiotherapy, chemotherapy, or pelvic contouring (6–8). The prognosis of cervical cancer is intricately linked to its clinical stage. The 5-year survival rate for early cervical cancer can surpass 90% (9); however, the rate for advanced, metastatic, or recurring CC is around 17% (10). The results of studies have demonstrated that minimally invasive or open pelvic contouring is a viable option for patients with advanced or recurrent disease. Furthermore, there is no notable discrepancy in survival analysis between the two approaches (11). The overall survival rate at five years for this procedure can reach as high as 40%; however, the incidence of serious complications is 22-32% (12), emphasizing the necessity for research into new, safe, and effective treatments.

The programmed cell death 1 (PD-1) receptor functions as an immunological checkpoint, but the programmed cell death ligand 1 (PD-L1) is often expressed on neoplastic cells. The link between PD-1 and PD-L1 allows tumor cells to circumvent immune monitoring. The inflammatory response, usually triggered by T cells in reaction to antigens, is terminated when the PD-1 receptor on the T cell interacts with PD-L1 on the host cell. This mechanism functions as an adaptive safeguard against extensive autoimmune reactions. Nonetheless, certain malignancies exploit this process by overexpressing PD-L1, therefore obstructing the immune system’s capacity to efficiently eliminate malignant cells (13). Currently, multiple clinical trials have evaluated the efficacy and safety of PD-1/PD-L1 immune checkpoint inhibitors (ICIs) in patients with diverse tumor types (14). PD-L1 expression has been shown in 34.4% to 96% of CC cases, whereas normal cervical tissues demonstrate little or no PD-L1 expression (15). Keynote-028 established that pembrolizumab is both safe and effective for the treatment of CC (16). As a result, the United States Food and Drug Administration (FDA) approved its use for PD-L1-positive recurrent or metastatic CC with disease progression during or after chemotherapy. Camrelizumab is a humanized high-affinity IgG4-kappa monoclonal antibody targeting programmed cell death 1 (PD-1) (17). Upon administration, the antibody binds to and obstructs the interaction of PD-1 with its ligands, programmed cell death ligand 1 (PD-L1), inhibits the activation of PD-1 and its downstream signaling pathways, and reinstates immune function by activating cytotoxic T lymphocytes and cell-mediated immune responses against tumor cells or pathogens. Activated PD-1 inhibits T-cell activation and is crucial in tumor escape from host immunity (18). As of yet, over 10 clinical studies or case series have released final or preliminary data concerning the effectiveness of Camrelizumab in patients with advanced CC. Of these studies, one exclusively addressed locally advanced CC, whereas the other nine examined advanced, recurring, or metastatic CC patients. The effectiveness of Camrelizumab for advanced CC treatment has been examined; nevertheless, the quantity of research is restricted, especially with a lack of high-quality randomized controlled trials. A comprehensive review and meta-analysis on Camrelizumab for CC is absent. This meta-analysis assesses the efficacy and safety of Camrelizumab in the treatment of CC, offering doctors recommendations for optimum clinical decision-making.

## 2 Materials and methods

### 2.1 Search strategy

A systematic search was conducted to retrieve published literature from PubMed, Embase, Web of Science, Cochrane Library, Ovid Medline, Scopus, ProQuest, China National Knowledge Infrastructure (CNKI), China Biology Medicine (CBM), Wan Fang, and VIP Database for Chinese Technical Periodicals (VIP) up to October 24, 2024. This meta-analysis imposed no language restrictions. The subject terms used in PubMed were Uterine Cervical Neoplasms [Mesh] and camrelizumab [Mesh]. The detailed search strategy is shown in **Table 1**.

**Table 1:** Search strategy in PubMed.

Number	Query	Results
#1	Search: “Uterine Cervical Neoplasms”[Mesh] Sort by: Most Recent	88,867
#2	Search: (((((((((((((((((((((Cervical Neoplasm, Uterine[Title/Abstract]) OR (Neoplasm, Uterine Cervical[Title/Abstract])) OR (Uterine Cervical Neoplasm[Title/Abstract])) OR (Neoplasms, Cervix[Title/Abstract])) OR (Cervix Neoplasm[Title/Abstract])) OR (Neoplasm, Cervix[Title/Abstract])) OR (Cervix Neoplasms[Title/Abstract])) OR (Cervical Neoplasms[Title/Abstract])) OR (Cervical Neoplasm[Title/Abstract])) OR (Neoplasms, Cervical[Title/Abstract])) OR (Cancer of the Uterine Cervix[Title/Abstract])) OR (Cancer of Cervix[Title/Abstract])) OR (Cancer of the Cervix[Title/Abstract])) OR (Cervix Cancer[Title/Abstract])) OR (Cancer, Cervix[Title/Abstract])) OR (Uterine Cervical Cancer[Title/Abstract])) OR (Cancer, Uterine Cervical[Title/Abstract])) OR (Cervical Cancer, Uterine[Title/Abstract])) OR (Uterine Cervical Cancers[Title/Abstract])) OR (Cervical Cancer[Title/Abstract])) OR (Cancer, Cervical[Title/Abstract])) OR (Cervical Cancers[Title/Abstract])	94,751
#3	#2 OR #1	119,462
#4	Search: “camrelizumab” [Supplementary Concept] Sort by: Most Recent	309
#5	Search: ((SHR-1210[Title/Abstract]) OR (SHR 1210[Title/Abstract])) OR (camrelizumab [Title/Abstract])	59
#6	#4 OR #5	337
#7	#3 AND #6	6

### 2.2 Study selection

The inclusion criteria for this meta-analysis were as follows: (1) Participants were individuals diagnosed with cervical cancer confirmed by pathological examination; (2) Interventions included either camrelizumab monotherapy or a combination of camrelizumab with other treatments; (3) The studies included in this analysis are of the following types: randomized controlled trials (RCTs), single-arm trials, prospective or retrospective cross-sectional cohort studies, and case-control studies. The following studies have been excluded: case reports, *in vitro* experiments, reviews, abstracts, letters, and pathological studies. Duplicate studies and those encompassing other tumors, from which data could not be collected independently, were excluded from the selection.

Following a collaborative conversation, Dr. Xiaodong Mi (MXD) and Dr. Fei Tuo (TF) executed the preliminary screening of the titles and abstracts of all acquired studies in alignment with the established screening approach. Individuals who did not satisfy the inclusion requirements were later excluded. MXD and TF subsequently conducted the independent extraction of information and data from research that satisfied the inclusion criteria. All differences were addressed through dialogue with a third researcher, Dr. Tong Lin (LT). The studies’ features include authors’ names, institutions, year of publication, research type, number of cases, patient age, camrelizumab dosage, combination with other medications, and outcome factors.

### 2.3 Outcome definitions

Outcome definitions included complete response (CR), partial response (PR), stable disease (SD), disease progression (PD), objective response rate (ORR), disease control rate (DCR), overall survival (OS), progression-free survival (PFS), and adverse events (AE). All adverse reactions are classified into grades 1–2 and 3–4, including anemia, increased aspartate aminotransferase, neutropenia, thrombocytopenia, fatigue, pain, hypertension, hand-foot syndrome, rash, diarrhea, anorexia, weight loss, pneumothorax, wound healing problems, oral mucositis, proteinuria, etc.

### 2.4 Quality assessment

Two of the three authors (MXD and TF) are to utilize the Risk of Bias in Non-randomized Studies of Interventions (ROBINS-I) to analyze the literature. ROBINS-I comprises seven assessment domains, each containing a series of inquiries, with responses categorized as “yes,” “probably,” “probably not,” “no,” and “no information.” Ultimately, a comprehensive evaluation of the risk level for each domain is provided, classified as “low risk,” “moderate risk,” “serious risk,” or “borderline risk.” The risk levels for all seven domains were acquired. The weighted Cohen’s kappa coefficient (κ) was used to evaluate the consistency of the evaluation outcomes between the two authors.

### 2.5 Statistical analysis

This meta-analysis utilized R version 4.4.1. A single-arm meta-analysis was conducted to assess the efficacy and incidence of adverse events in patients receiving camrelizumab treatment. A generalized linear mixed model was utilized through the metaprop function, with interval estimation performed using the Clopper-Pearson interval method. When proportions were either 0 or 1, the PFT sampling technique was utilized; in other cases, PRAW was chosen. The I² statistic and Cochran’s Q test were employed to evaluate statistical heterogeneity. This study utilized a random-effects model for analysis, irrespective of heterogeneity levels, due to prior single-arm meta-analyses indicating I² values often surpassing 90% and recent findings suggesting that random-effects models yield more reliable outcome estimates compared to fixed-effects models (19). The combined results were illustrated in forest plots, and Egger’s test was conducted with thresholds of P < 0.01, P > 0.01, and P < 0.01, respectively. A p-value less than 0.05 signifies the presence of significant publication bias.

## 3 Results

### 3.1 Selection of literature and quality assessment

**Figure 1:**
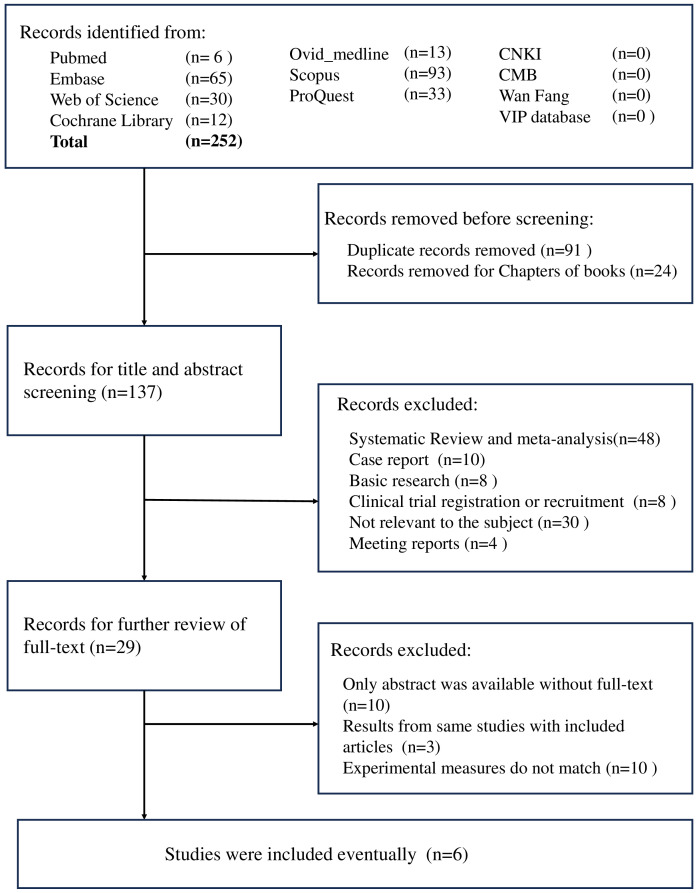
Research screening flowchart.

A total of 252 studies were identified in our initial search. After the exclusion of repetitive studies and book chapters, 137 studies remained. Following the screening of titles and abstracts, we excluded 48 systematic reviews and meta-analyses, 10 case reports, 8 basic research articles, 8 clinical trial recruitment studies, 4 meeting reports, and 30 irrelevant studies. Twenty-nine conference papers were excluded after a thorough review of the full text (**Figure 1**). A total of six studies involving 238 patients with CC were included in this meta-analysis. **Table 2** presents the information contained in each study. The “Zhang X 2022” study involved the treatment of 35 patients with camrelizumab, with safety data recorded for each individual. Furthermore, two patients were lost to follow-up, while 25 patients received treatment as per the protocol for efficacy analysis.

**Table 2:** Characteristic of included studies.

First author(year)	Nation	Registration Number	Trial phase	Intervention	Camrelizumab Does	Sample size	Mean age, years	Endpoints
Lan C 2020 (28)	China	NCT03816553	Single-Arm Phase II Trial	Camrelizumab+ apatinib	200 mg q2w	45	51 (33–67)	①②③④⑤⑥⑦⑧⑨
Zhang X 2022 (29)	China	ChiCTR1900025992	Single-Arm Phase II Trial	Camrelizumab+ chemotherapy	200 mg q3w	35	50(30-71)	①②③④⑤⑥⑧
Xia L 2022 (32)	China	NCT03827837	Single-Arm Phase II Trial	Camrelizumab+famitinib	200 mg q3w	33	50(43-55)	①②③④⑤⑥⑦⑧
Li G 2023 (33)	China	NA	Retrospective Study	Camrelizumab+ chemotherapy+ apatinib	200 mg q3w	15	50 (30–71)	①②③④⑤⑥⑦⑧⑨
Li K 2024 (30)	China	NCT04516616	Single-Arm phase 2 trial	Camrelizumab+ chemotherapy	200 mg q3w	85	51(46-57)	①②⑤⑧
Jian X 2024 (34)	China	NA	Retrospective Study	Camrelizumab+ chemotherapy	200mg	25	51.0 ± 1.8	①②③④⑤⑥⑧

①CR; ②PR; ③SD; ④PD; ⑤ORR; ⑥DCR; ⑦OS; ⑧;PFS; ⑨AEs.

Two of the three authors (MXD and TF) conducted an independent assessment of the quality of all articles in this review using the Cochrane risk of bias tool, using the weighted Cohen kappa coefficient (κ) to quantify agreement. The final Cohen kappa coefficient of bias was determined to be 1 (p < 0.001), signifying that the agreement between the two assessments was nearly perfect. The results of the assessments are presented in **Table 3** following discussions among the three researchers.

**Table 3:** Quality assessment of the non-randomized controlled studies (ROBINS-I).

Study	Bias due to confounding	Bias in selection of participants into the study	Bias in classification of interventions	Bias due to deviations from intended interventions	Bias due to missing data	Bias in measurement of outcomes	Bias in selection of the reported result	Overall bias
Lan C 2020	Low	Low	Low	Low	Low	Low	Low	Low
Zhang X 2022	Low	Low	Low	Low	Serious	Low	Low	Serious
Xia L 2022	Low	Low	Low	Low	Low	Low	Low	Low
Li G 2023	Low	Low	Low	Low	Moderate	Low	Low	Moderate
Li K 2024	Low	Low	Low	Low	Low	Low	Low	Low
Jian X 2024	Low	Moderate	Low	Low	Low	Low	Low	Moderate

### 3.2 Therapeutic efficacy assessments

**Figure 2:**
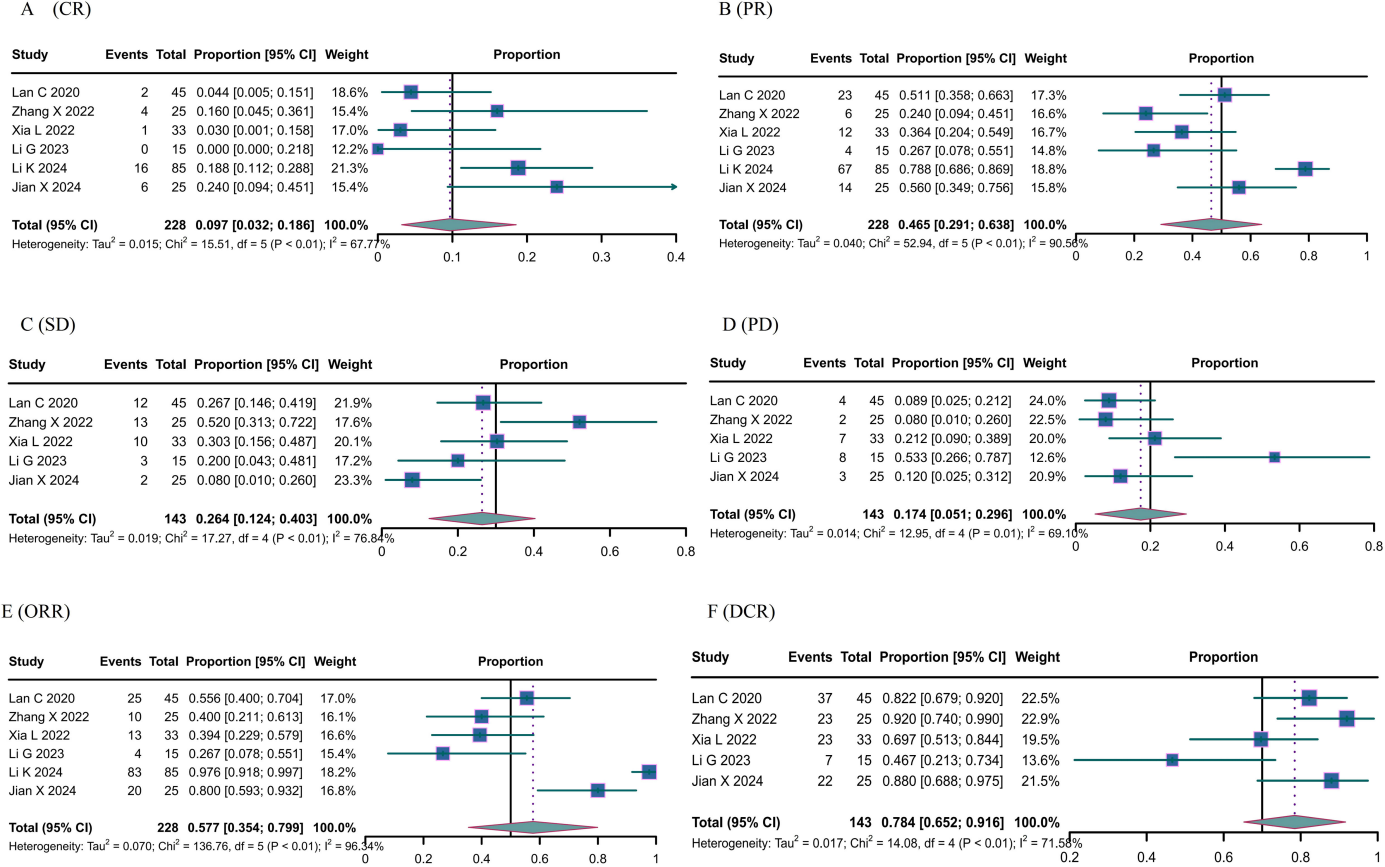
The response of Canrelizumab for the treatment of cervical cancer. **(A)** complete response, **(B)** partial response, **(C)** stable disease, **(D)** disease progression, **(E)** objective response rate, and **(F)** disease control rate.

All studies included in the analysis reported the efficacy response of camrelizumab in cervical cancer. Due to the predominance of single-arm studies and notable heterogeneity among them, a random-effects model was employed. In a cohort of 228 patients, 29 achieved complete response (CR) with a proportion of 0.097 (95% CI: 0.032-0.186) as illustrated in **Figure 2A**. Additionally, 126 patients attained CR, resulting in a proportion of 0.465 (95% CI: 0.291-0.638) shown in **Figure 2B**. Among 143 patients, 40 achieved stable disease (SD), corresponding to a proportion of 0.264 (95% CI: 0.124-0.403) depicted in **Figure 2C**, while 24 patients experienced progressive disease (PD), with a proportion of 0.174 (95% CI: 0.051-0.296) represented in **Figure 2D**. Six studies documented the overall response rate (ORR), while five studies documented the disease control rate (DCR). The objective response rate (ORR) was 0.577 (95% confidence interval: 0.354-0.799) (**Figure 2E**), while the disease control rate (DCR) was 0.784 (95% confidence interval: 0.652-0.916) (**Figure 2F**). The original data is accurate; therefore, the combined effect of OS and FPS is not computed.

### 3.3 AEs assessments

**Figure 3:**
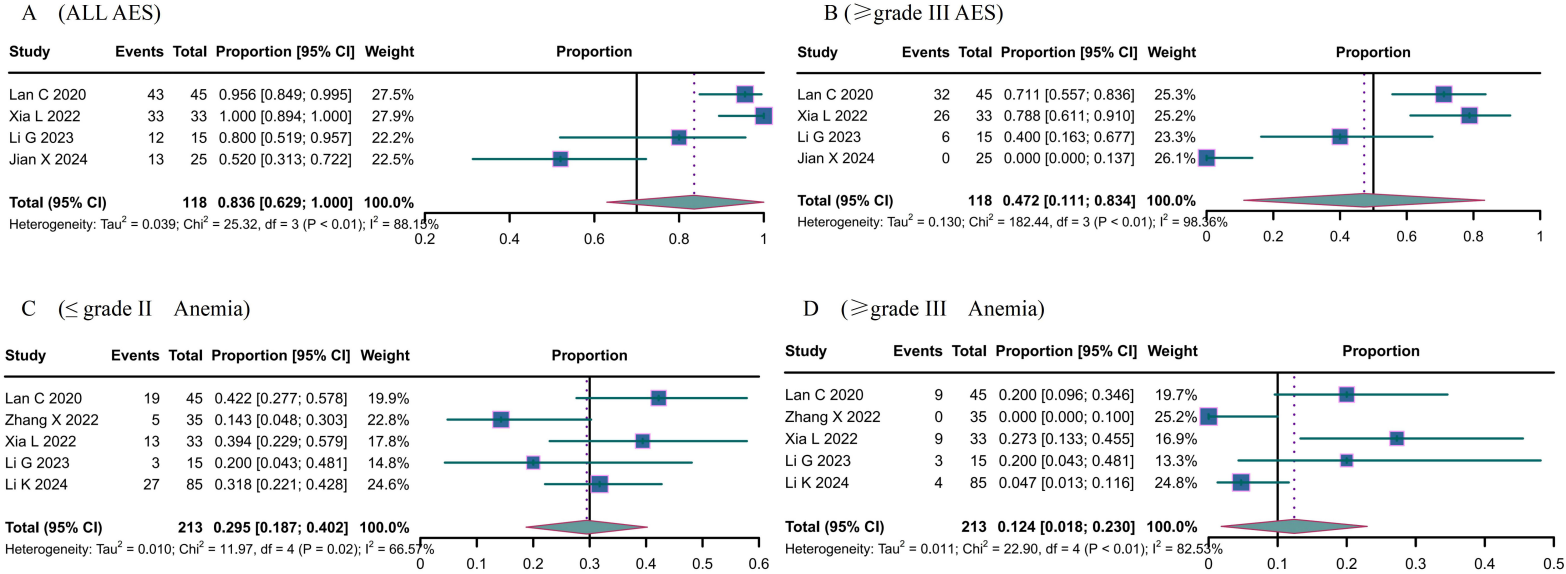
The Adverse events (AE) of Camrelizumab for the treatment of cervical cancer. **(A)** all AES, **(B)** ≥ grade III AES, **(C)** ≤ grade II Anemia, **(D)** ≥ grade III Anemia.

Adverse events primarily consisted of anemia, abnormal liver function, neutropenia, thrombocytopenia, hypertension, fatigue, hypothyroidism, and nausea and vomiting. The overall incidence of adverse events was 0.836 (95% CI 0.629-1.000) (**Figure 3A**); grade ≥ III adverse events was 0.472 (95% CI 0.111; 0.834) (**Figure 3B**). The most prevalent treatment-related adverse events included anemia [≤grade II: 0.295, 95% CI: 0.187-0.402 (**Figure 3C**); ≥grade III: 0.124, 95% CI: 0.018-0.230 (**Figure 3D**)], increased aspartate aminotransferase (≤grade II: 0.196, 95% CI: 0.013-0.380; ≥grade III: 0.030, 95% CI: 0.007-0.053), neutropenia (≤grade II: 0.206, 95% CI: 0.150-0.261; ≥grade III: 0.114, 95% CI: 0.066-0.162), thrombocytopenia (≤grade II: 0.295, 95% CI: 0.187-0.402), and fatigue (≤grade II: 0.174, 95% CI: 0.046-0.303), among others.

## 4 Discussion

Cervical cancer represents one of the most prevalent tumors among women and is among the most preventable malignant tumors (5). The combination of human papillomavirus (HPV) and liquid-based cytology (TCT) screening, along with the administration of the HPV vaccine, has resulted in a notable reduction in the incidence and mortality rates of cervical cancer, particularly in countries with a high Human Development Index (HDI) (1). CC has the potential to be the first human malignancy eradicated. Despite these advancements, cervical cancer continues to account for 604,127 new cases and 341,830 deaths each year (1). CC remains a notable public health issue, especially in areas characterized by lower human development indices. A discernible trend toward a younger age of incidence is evident (20). The prognosis of cervical cancer is significantly associated with its clinical stage. The five-year survival rate for patients with early-stage cervical cancer may surpass 90% (9), while for those with advanced, metastatic, or recurrent cervical cancer, it is roughly 17% (10). The current first-line treatment for cervical cancer (CC) includes surgical, radiotherapeutic, and chemotherapeutic interventions (with or without the anti-VEGF drug bevacizumab) (21). The mortality rate of CC has been reduced as a result of these treatments; however, the prognosis for patients with advanced, recurrent, or metastatic CC remains poor. Furthermore, the current treatment regimens are frequently associated with adverse events that result in a decline in the patient’s quality of life and even necessitate treatment interruptions. It is therefore imperative to investigate novel clinical treatment modalities with the objective of enhancing therapeutic efficacy and prolonging overall survival in patients.

Typically, the immune system within the body maintains a dynamic equilibrium between activation and suppression. Immune checkpoint inhibitors (ICIs) represent a novel therapeutic approach that has emerged in recent years. It mobilizes the immune system to combat tumor cells by eliminating immunosuppression within the immune microenvironment. B7 is expressed on T cells within the immune system and can bind to MHC I molecules on antigen-presenting cells, thereby activating T cells and inducing an immune response (22). The programmed death receptor 1 ligand (PD-L1), which is found on the surface of antigen-presenting cells or tumor cells, may bind to the programmed death receptor 1 (PD-1) on T cells, which stops T cells from working and the immune system from working altogether (23). When PD-L1 expression is elevated on tumor cells, this results in the suppression of the immune microenvironment, which in turn leads to the suppression of T cell function and the failure to clear tumor cells. By blocking the PD-1/PD-L1 pathway, ICIs suspend immunosuppression and reactivate the immune system, thereby eliminating tumor cells (24).

The advent of immunotherapy has established itself as a pivotal therapeutic approach following surgical, radiotherapeutic, chemotherapeutic, and targeted therapeutic interventions. Its emergence has provided a new direction for the treatment of patients with advanced, recurrent, or metastatic malignancies. At present, ICIs are the most commonly used monoclonal antibodies in clinical practice, targeting PD-1, PD-L1, and cytotoxic T-lymphocyte-associated antigen-4 (CTLA-4) (25). A substantial body of clinical evidence has demonstrated the potential benefits of ICIs in the treatment of various malignancies, including non-small cell lung cancer (NSCLC), melanoma, and kidney cancer. The Phase II KEYNOTE-158 study enrolled 98 patients with locally advanced or metastatic cervical cancer who were treated with pembrolizumab (200 mg every three weeks). The objective response rate (ORR), median progression-free survival (mPFS), and median overall survival (mOS) were 12.2% (95% CI, 6.5-20.4%), 2.1 months (95% CI, 2.0-2.2 months), and 9.4 months (95% CI, 7.7-13.1 months), respectively (26). The CheckMate 358 trial, a phase I/II, single-arm, and open-label study, included 19 patients with recurrent/metastatic cervical cancer who were treated with nivolumab (240 mg every fortnight). The study results demonstrated ORR and DCR of 26.3% (95% CI, 9.1-51.2%) and 68.4% (95% CI, 43.4-87.4%), respectively. The mPFS, mOS, and 12-month overall survival (OS) rates were 5.1 months (95% CI, 1.9-9.1 months), 21.9 months (95% CI, 15.1 months-NR), and 77.5% (95% CI, 50.5-91.0%), respectively (27). Furthermore, combination therapy has been demonstrated to enhance clinical efficacy while reducing adverse effects and offering patients a more tailored and comprehensive treatment plan. A multicenter, open-label, single-arm, Phase II trial (NCT03816553) enrolled 45 patients with advanced, recurrent, or metastatic cervical cancer. These patients were treated with camrelizumab (200 mg every 2 weeks) and apatinib (250 mg once per day). The results of the study demonstrated that the ORR and DCR were 55.6% (95% CI, 40.0-70.4) and 82.2% (95% CI, 70.6-93.8), respectively. Additionally, the mPFS and mOS were 20.3 months (95% CI, 9.3-36.9) and 8.9 months (95% CI, 5.6-18.1), respectively (28).

Camrelizumab is a monoclonal antibody engineered to obstruct the connection between the programmed cell death protein 1 (PD-1) receptor and its ligand, PD-L1. Camrelizumab inhibits the interaction between PD-1 and PD-L1 by binding to PD-1, hence obstructing the activation of PD-1 and its downstream signaling pathways. This inhibition reinitiates immune responses by activating cytotoxic T lymphocytes and enhancing a cell-mediated immune response against neoplastic cells or pathogens. Activation of PD-1 is recognized to have an inhibitory influence on T cell activation, hence promoting tumor immune evasion and enabling malignancies to circumvent host immunological defenses (18). Camrelizumab received its first global approval in China on 31 May 2019 for the treatment of patients with relapsed or refractory classical Hodgkin’s lymphoma who have received at least two prior systemic chemotherapy treatments (18). More clinical trials have confirmed the efficacy of camrelizumab in non-squamous non-small cell lung cancer, nasopharyngeal carcinoma, hepatocellular carcinoma, and gastric and gastroesophageal junction cancer (18, 29). The inaugural recorded application of camrelizumab in gynecological oncology transpired in 2020, when Lan C employed camrelizumab for the management of advanced, recurring, or metastatic cervical cancer (28). In 2024, Li K employed camrelizumab as neoadjuvant chemotherapy for locally advanced cervical carcinoma (30). An increasing volume of data suggests the efficacy of camrelizumab in treating individuals with various stages of cervical cancer. A deficiency of meta-analyses exists that consolidate these trials to elucidate the effectiveness and safety of camrelizumab in the treatment of cervical cancer.

This meta-analysis evaluated the effectiveness and safety of camrelizumab for cervical cancer therapy, involving 238 patients across six trials. The effectiveness was evaluated in 228 individuals, while safety was tested in all patients. Pooled evaluations indicated that camrelizumab had effectiveness and a tolerable safety profile in the treatment of cervical cancer. Favorable clinical responses, including complete response (CR), partial response (PR), stable disease (SD), overall response rate (ORR), and disease control rate (DCR), were noted despite variations among patients concerning illness stage, kind, and prior therapy. This meta-analysis reported full remission rates of 9.7%, partial remission rates of 46.5%, stable disease rates of 26.4%, disease progression rates of 17.4%, objective remission rates of 57.7%, and disease control rates of 78.4%. Five trials involved patients with advanced, recurrent, or metastatic cervical cancer, whereas one research study included patients with locally advanced cervical cancer; hence, SD, PD, and DCR were absent from the efficacy evaluation metrics in Li K’s study during the efficacy assessment. To mitigate the bias of this meta-analysis, the subgroup analysis was conducted subsequent to the exclusion of Li K’s paper. The findings indicated a complete remission rate of 7.6%, a partial remission rate of 39.2%, and an objective remission rate of 49.2%, reflecting a little decline in CR, PR, and ORR relative to the aggregated results of the six investigations. Pooled analyses of overall survival (OS) and progression-free survival (PFS) were not feasible due to the unavailability of OS and PFS data in certain included studies, inadequate median OS (mOS)/median PFS (mPFS) follow-up periods, and the reporting of progression-free survival at 6, 9, and 12 months instead of mOS and mPFS. The minimum mPFS was 3.0 months, and the maximum median PFS was 10.3 months. The minimum mOS was 8.0 months, and the maximum mOS was 20.3 months. **Table 4** illustrates the findings of this study in comparison with the meta-analysis that assesses the efficacy and safety of pembrolizumab on cervical cancer (31). The single-arm meta-analysis demonstrated a complete response rate of 2.7%, a partial response rate of 10.4%, a stable disease rate of 19%, a progressive disease rate of 54.1%, and an objective response rate of 15.5%. The disease control rate was 33.1%, with a mOS of 10.23 months, a mPFS of 4.27 months, a best response time of 2.1 months, and a one-year mortality rate of 38.8%. Upon evaluating just the CR, PR, SD, PD, ORR, and DCR data, it may be surmised that the therapeutic efficacy of camrelizumab surpasses that of pembrolizumab. The mOS and mPFS of patients administered pembrolizumab are situated between the shortest mOS and mPFS recorded in patients treated with camrelizumab and the longest mOS and mPFS seen (31). The outcomes of such comparative studies are dependent upon numerous variables. Therefore, to ascertain the efficacy of camrelizumab and pembrolizumab in the treatment of cervical cancer, a rigorous randomized controlled trial must be conducted.

**Table 4:** Comparison of outcomes between Camrelizumab and Pembrolizumab in the treatment of cervical cancer.

	Camrelizumab	Pembrolizumab
**Type of study**	Single-arm trial	Single-arm trial
**Number of studies included**	6	7
**Number of patients included**	238	727
**Combined outcome indicators**	CR, PR, SD, PD, ORR, DCR, ALL AES, ≥grade III AES, ≤grade II Anemia, ≥grade III Anemia	CR, PR, SD, PD, ORR, DCR, OS, PFS, TTR, The 1-year death rate, ≥grade III AES
	Proportions, 95% CI	Proportions, 95% CI
**CR**	0.097(0.032-0.186)	0.029(0.007-0.059)
**PR**	0.465(0.291-0.638)	0.108(0.069-0.163)
**SD**	0.264(0.124-0.403)	0.190(0.149-0.240)
**PD**	0.174(0.051-0.296)	0.541(0.421-0.661)
**ORR**	0.577(0.354-0.799)	0.155(0.098-0.236)
**DCR**	0.784(0.652-0.916)	0.331(0.277-0.385)
**PFS**	8.9(5.6-18.1)*	0.427(1.57-6.96)
**OS**	20.3(9.3-36.9)*	10.23(8.96-11.50)
**≥grade III AES**	0.472(0.111-0.834)	0.212(0.065-0.509)

*Pooled analyses of OS and PFS were not possible due to the lack of data on OS and PFS in some of the included studies, or the follow-up time for median OS/median PFS was not achieved, or the rate of progression-free survival at 6, 9, and 12 months was recorded; therefore, the results of median PFS and median OS in the study of Lan C 2020, for which complete data were available, were compared with those of Pembrolizumab Comparison.

Bold fonts indicate the primary comparison items.

Since the advent of immunotherapy, considerable attention has been devoted to the compliance and tolerability of these drugs, which are frequently associated with a range of adverse effects that can impede patients’ ability to adhere to or tolerate prolonged treatment, particularly during maintenance therapy, in older age groups, or in the presence of multiple comorbidities. The results of this meta-analysis demonstrated that the most prevalent adverse events associated with the use of camrelizumab were anemia, thrombocytopenia, neutropenia, impaired hepatic function, and fatigue. The majority of these adverse events were Grades 1-2. The combined effect size post-analysis yielded a prevalence of grade 1-2 adverse events (AEs) of 83.6%, grade 3-4 AEs of 47.2%, grade 1-2 anemia of 29.5%, grade 3-4 anemia of 12.4%, grade 1-2 aspartate aminotransferase (AST) elevation of 19.6%, and grade 3-4 AST elevation of The prevalence of grade 1-2 thrombocytopenia was 3.0%, while the prevalence of grade 1-2 neutropenia and grade 1-2 fatigue was 20.6% and 17.4%, respectively. By reviewing the literature, it can be seen that most of the patients can tolerate AEs after they occur and can be relieved after treatment with medication. The above results indicate that camrelizumab has a good safety profile in the treatment of cervical cancer, and the occurrence of AEs during treatment is manageable. This is similar to the results of other safety studies on PD-1/PD-L1 for cervical cancer.

It should be noted that this meta-analysis was subject to a number of limitations. Primarily, the number of studies included was relatively small, and the total number of participants was low. Furthermore, some eligible studies were excluded because the original articles could not be obtained through additional database searches and attempts to contact the authors, and therefore these studies for which the original articles could not be obtained were not included in the analysis. Secondly, the included literature demonstrated significant heterogeneity, which was primarily attributable to the inconsistency in the drugs with which camrelizumab was combined across the studies. The combination therapy in Lan C’s trial was apatinib (28), in Zhang X’s study it was albumin paclitaxel combined with carboplatin (29), and in Xia L’s study it was famitinib (32). Subgroup analysis of the trials was infeasible due to an insufficient number of trials utilizing the same medication combinations. Thirdly, due to the trials being only single-arm or retrospective studies without controlled trials, we can only evaluate effectiveness and risk and cannot definitively conclude if therapy with camrelizumab is advantageous.

## 5 Conclusion

In conclusion, the meta-analysis demonstrated the efficacy and safety of camrelizumab in the treatment of cervical cancer, providing evidence for its future clinical application. Nevertheless, given the restricted number of clinical studies and the limited number of patients included, it is imperative that large-scale, multicenter, cross-border, and cross-race randomized controlled trials be conducted in the future to validate this conclusion.

## Data Availability

The original contributions presented in the study are included in the article/supplementary material. Further inquiries can be directed to the corresponding author.
